# Ramsay Hunt Syndrome With Cranial Polyneuropathy and Delayed Facial Nerve Palsy: A Case Report

**DOI:** 10.7759/cureus.27434

**Published:** 2022-07-29

**Authors:** Raid M Al-Ani

**Affiliations:** 1 Department of Surgery, Otolaryngology, College of Medicine, University of Anbar, Ramadi, IRQ

**Keywords:** case report, ramsay hunt syndrome, facial nerve paralysis, polyneuropathy, herpes zoster, herpes zoster oticus

## Abstract

Herpes zoster oticus is a viral disease caused by the reactivation of the varicella-zoster virus at the geniculate ganglion. The hallmark of the condition is multiple unilateral erythematous vesicles, which are distributed over the auricle and preceded by severe otalgia. If these symptoms are associated with facial nerve palsy, the condition is called Ramsay Hunt syndrome (RHS) which is usually accompanied by vestibulocochlear abnormalities. A 42-year-old woman came to our clinic with sudden onset of right-sided severe otalgia and several erythematous vesicles on the auricle two days ago. She provided a history of dysphagia and hoarseness for 10 days. After two days, ipsilateral facial nerve paralysis was noted. The patient was immunocompetent with an unremarkable medical history. Physical examination revealed the following: the vesicles distributed over the right auricle, external auditory canal, and eardrum; right sensorineural deafness; deviated uvula to the left side; absent gag reflex on the right side; right vocal cord palsy; and lower motor facial nerve paralysis of House-Brackmann grade III. The pure tone audiogram confirmed the diagnosis of right-sided sensorineural deafness. Acyclovir therapy and prednisolone tablets at a loading dose were initiated. At the four-month follow-up, the presenting manifestations were improved. Here, we report a case of RHS with early glossopharyngeal and vagus nerve palsy, followed by pain, vesicular eruptions, sensorineural hearing loss, and delayed onset of facial nerve paralysis. The condition resolved completely on medical treatment with acyclovir and prednisolone.

## Introduction

Herpes zoster oticus (HZO) is a rare clinical entity encountered in clinical practice. It is a viral disease caused by the reactivation of the varicella-zoster virus (VZV) at the geniculate ganglion. HZO is characterized by unilateral vesicular eruptions over the pinna, external ear canal, and tympanic membrane which is preceded by severe otalgia. If the condition is associated with ipsilateral lower motor facial palsy, it is called Ramsay Hunt syndrome (RHS). This syndrome is frequently associated with vestibulocochlear manifestations. However, other cranial nerve involvement is extremely rare [1,2]. Delayed facial nerve paralysis is seldom seen in the Otology practice.

A recent study from Korea reported a case of RHS with a delayed onset of the trigeminal and vagus nerve palsy 16 days after the involvement of the trigeminal, facial, and vestibulocochlear nerves [3]. In addition, two cases of delayed onset VII nerve paralysis in patients with RHS were also reported from Korea [4]. To our knowledge, there has been no reported case of RHS with early glossopharyngeal and vagus nerve palsy, followed by pain, vesicular eruptions, sensorineural hearing loss, and a delayed onset facial nerve paralysis. Hence, we present the first case with a non-classical presentation similar to the above-mentioned presentation.

## Case presentation

A 42-year-old woman presented at the Otolaryngology clinic with a sudden onset of dysphagia, odynophagia, and hoarseness 10 days ago. One week after these symptoms, she complained of right-sided sudden deafness, severe otalgia, and erythematous vesicular eruptions over the right pinna. Two days later, she developed right-sided facial palsy. She was healthy before these symptoms. The patient’s medical history was unremarkable. Neither the patient nor her family had a similar problem.

**Figure 1:**
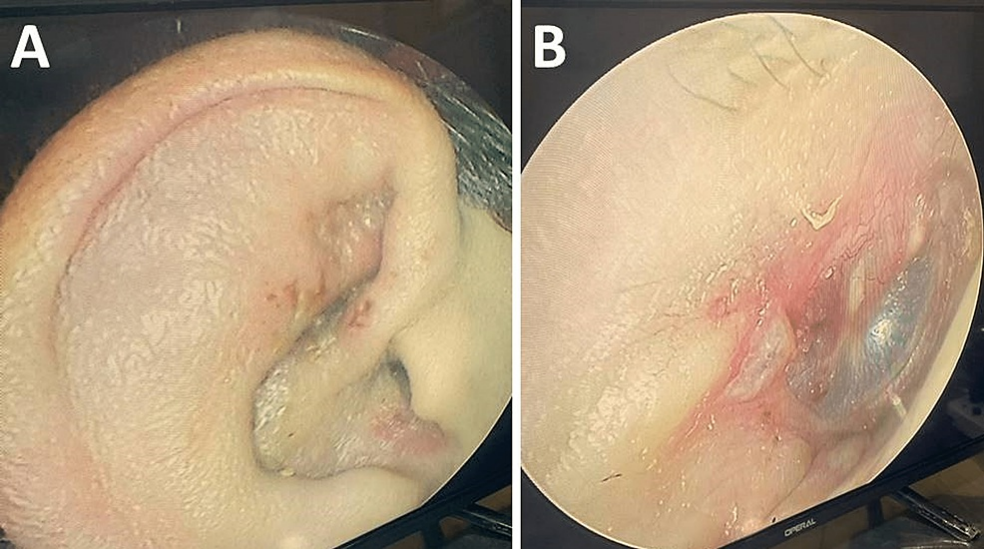
Vesicular eruption of the right auricle (A), external auditory canal, and tympanic membrane (B).

**Figure 2:**
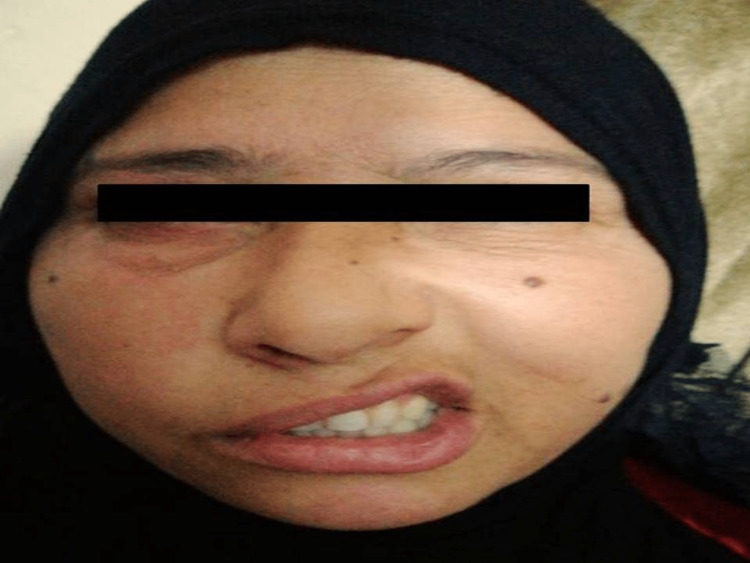
Right lower motor neuron facial nerve paralysis includes flattening of the nasolabial fold, drop of the oral angle, and reduced wrinkles on the movement of the forehead.

Physical examination revealed erythematous vesicular eruptions over the right auricle, external auditory canal, and eardrum (Figure 1). Tuning fork tests showed that the Rinne test was positive on both sides, the Weber test lateralized to the left ear, and the absolute bone conduction test reduced on the right side. Oropharyngeal examination revealed that the uvula deviated to the left side on phonation and reduced gag reflex on the right side. Laryngoscopic examination showed immobility of the right vocal cord. In addition, there was a complete right lower motor neuron facial nerve palsy (Figure 2).

**Figure 3:**
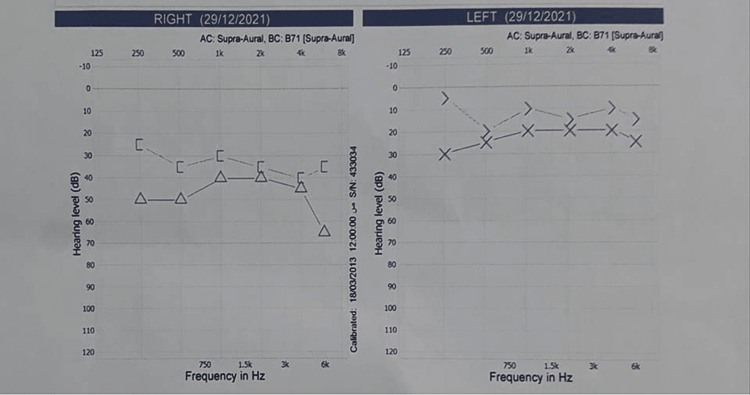
A pure tone audiogram showing right-sided sensorineural hearing loss of 43 dB.

Blood tests revealed that the erythrocyte sedimentation rate (ESR) was 25 mm/hour (reference range = 0-15 mm/hour), and C-reactive protein was 12 mg/dL (reference range negative <6 mg/dL). There were positive immunoglobulin (Ig)G antibodies against VZV and negative IgM antibodies against VZV on serological tests. Both IgG and IgM antibodies against severe acute respiratory syndrome coronavirus 2 (SARS-CoV-2) were negative, as well as there were no abnormal routine blood tests. A pure tone audiogram showed right-sided sensorineural hearing loss (43 dB hearing loss), as shown in Figure 3. There were no signs of intracranial neoplasm, infection, or demyelinating abnormalities on magnetic resonance imaging (MRI) of the brain.

The patient was diagnosed with RHS with polyneuropathy. She was hospitalized and a loading dose of 1 mg/kg/day of prednisolone orally was started in three divided doses. Subsequently, gradual tapering was done over three weeks. In addition, intravenous acyclovir was given at a dose of 25 mg/kg/day for 10 days. Dressing of the ear on a daily basis was performed and a pain killer was given. Physiotherapy of the patient was initiated in the rheumatology and rehabilitation unit. No side effects were detected during or following the completion of the treatment regimen.

After two weeks of follow-up, the vesicles dried up changed to scabs and dropped off. Her hearing impairment fully recovered with partial improvement of facial movement. At four months of follow-up, there were complete normal functions of the facial, glossopharyngeal, and vagus nerves.

## Discussion

RHS has an incidence of 5/100,000 individuals/year. The syndrome accounts for about 7% of the cases with acute seventh cranial nerve palsy [5]. Although RHS mostly presents in individuals aged 60-80 years, any age from 19 to 89 years can be involved [2]. It affects any person, whether with a normal or depressed immune system. However, the more severe disease form and less favorable outcomes occur in patients with hypo-immunity. There are many predisposing factors that can increase the incidence of RHS, such as stress, infection, malnutrition, cytotoxic drugs, diabetes mellitus, and malignant tumors.

The classical presentation of RHS is a triad of unilateral severe otalgia which is preceded by the appearance of erythematous vesicular lesions over the auricle, external ear canal and eardrum, and lower motor neuron facial nerve paralysis. The vestibulocochlear nerve is the most common cranial nerve associated with the syndrome [2]. The oculomotor, trigeminal, abducent, glossopharyngeal, and vagus nerves are also associated with RHS also reported in the literature as RHS with polyneuropathy [4,6,7]. Moreover, non-classical presentations such as RHS without the involvement of the facial nerve have also been reported [8]. There are five possible mechanisms of simultaneous cranial polyneuropathy, namely, VZV induces occlusive vasculitis which results in ischemic neuropathy, the spread of the infection through the synapse, a neuroinvasive feature of the virus, inflammation of contiguous ganglia, and anastomoses between the affected nerves and other cranial nerves [9,10].

Our patient presents another non-classical presentation of the RHS. The presenting case complained of sudden-onset dysphagia and hoarseness. One week later, she complained of vesicular eruptions in the right auricle, which was preceded by severe otalgia and deafness. Two days later, she developed right-sided facial palsy.

The diagnosis of RHS is usually based on the classical presentation of the syndrome (unilateral severe earache, vesicular rash over the pinna, facial nerve palsy, and in most cases sensorineural hearing loss) [11]. Confirmation of the diagnosis is carried out with serological tests of IgG and IgM antibodies against VZV. Brain MRI is mandatory for the exclusion of tumors or demyelinated lesions or when there is a suspicion of infective brain tissue complications of the VZV. The presenting case revealed a completely normal brain MRI.

The novel coronavirus disease 2019 (COVID-19) can suppress the immune system and thereby can increase the risk of bacterial or viral diseases including HZO. RHS might be the presenting or the only feature of COVID-19. Therefore, it is crucial to test patients to rule out or confirm SARS-CoV-2 even if there are no other features of COVID-19 [12]. Because the presenting case was detected in the era of COVID-19, she was sent for serological testing. The test results excluded the association between the RHS and COVID-19.

Although RHS is considered to resolve without treatment, early initiation of treatment is advised to reduce long-term complications such as postherpetic neuralgia and spastic facial nerve palsy. Several investigations have reported a significant reduction in late complications with the combination of steroid and antiviral therapy [13,14]. Our patient also showed complete improvement with these two treatment modalities.

The prognosis of RHS is less favorable than Bell’s palsy [14]. The full recovery rate was reported to be 63.63% in patients with an RHS with cranial polyneuropathy [7]. Furthermore, the prognostic factor that seems as reported in the literature is the severity of the presenting features [13]. Even though there was slow recovery of the involved cranial nerves in the presenting case, complete recovery was achieved at the four-month follow-up.

## Conclusions

RHS is a rare condition and non-classical presentation is seldom seen in clinical practice. This is another case of RHS with an atypical presentation. High suspicion is necessary to diagnose the syndrome as early as possible and treat it accordingly with steroid and antiviral therapy to prevent late complications such as postherpetic neuralgia.
